# Postpartum Circulating Cell-Free Insulin DNA Levels Are Higher in Women with Previous Gestational Diabetes Mellitus Who Develop Type 2 Diabetes in Later Life

**DOI:** 10.1155/2019/3264184

**Published:** 2019-07-24

**Authors:** Martha Lappas, Harry M. Georgiou, Jane C. Willcox, Michael Permezel, Alexis Shub, Cody-Lee Maynard, Mugdha V. Joglekar, Anandwardhan A. Hardikar

**Affiliations:** ^1^Obstetrics, Nutrition and Endocrinology Group, Department of Obstetrics and Gynaecology, University of Melbourne, Mercy Hospital for Women, Heidelberg, Victoria, Australia; ^2^Department of Obstetrics and Gynaecology, University of Melbourne, Mercy Hospital for Women, Heidelberg, Victoria, Australia; ^3^School of Allied Health, College of Science, Health and Engineering, La Trobe University, Bundoora, Victoria, Australia; ^4^Islet Biology and Diabetes Group, NHMRC Clinical Trials Centre, The University of Sydney, Camperdown, NSW, Australia

## Abstract

**Background:**

Women with previous gestational diabetes mellitus (GDM) have evidence of postpartum *β*-cell dysfunction, which increases their risk of developing type 2 diabetes (T2DM) later in life. Elevated levels of circulating cell-free preproinsulin (*INS*) DNA correlate with dying *β*-cells in both mice and humans. The aim of this study was to determine if cell-free circulating *INS* DNA levels are higher in women with previous GDM who develop T2DM.

**Methods:**

We used droplet digital (dd) PCR to measure the levels of cell-free circulating methylated and unmethylated *INS* DNA in plasma from 97 women with normal glucose tolerance (NGT), 12 weeks following an index GDM pregnancy. Women were assessed for up to 10 years for the development of T2DM.

**Results:**

In the follow-up period, 22% of women developed T2DM. Compared with NGT women, total cell-free *INS* DNA levels were significantly higher in women who developed T2DM (*P* = 0.02). There was no difference in cell-free circulating unmethylated and methylated *INS* DNA levels between NGT women and women who developed T2DM (*P* = 0.09 and *P* = 0.07, respectively).

**Conclusions:**

In women with a previous index GDM pregnancy, postpartum levels of cell-free circulating *INS* DNA are significantly higher in those women who later developed T2DM.

## 1. Introduction

Type 2 diabetes (T2DM) is a major cause of morbidity and mortality worldwide, and the incidence is increasing, especially in low- and middle-income countries [1, 2]. Gestational diabetes mellitus (GDM) is an important predictor for the development of T2DM in later life [3]. The early identification of those at greatest risk of developing T2DM may assist in providing targeted lifestyle intervention and/or medications to delay the development of T2DM [4].

Cell-free circulating DNA (cfDNA), derived from dead cells and detectable in plasma and serum, is now recognized as a potential biomarker for a variety of diseases including cancer [5], cardiovascular disease [6], and type 1 diabetes [7]. With respect to type 1 diabetes, cell-free circulating preproinsulin (*INS*) DNA has been commonly studied as a marker of pancreatic *β*-cell death. In humans, the insulin promoter is predominantly unmethylated in islet *β*-cells and methylated in all other tissues tested [8, 9]; however, studies in mice have shown that in response to immunological stressors, there is an increase in *β*-cell methylated *INS* DNA [10]. As such, elevated levels of methylated and/or unmethylated *INS* DNA in people with new-onset type 1 diabetes have been reported [11, 12]. Notably, prospective studies have shown that circulating unmethylated *INS* DNA is positively associated with the development of type 1 diabetes [13]. Experimental animal models of type 1 diabetes have also shown that significantly upregulated levels of circulating unmethylated *INS* DNA are detectable before the onset of hyperglycemia [9, 14, 15]. Very recently, in islet transplant recipients, *β*-cell death estimation using insulin cfDNA is shown to correlate with clinical islet transplantation outcome [16].

When compared to normal glucose tolerant (NGT) women, women with GDM have decreased *β*-cell function 5 years postpartum [17] which may be linked to the increased risk of developing T2DM later in life [3]. In this study, we used droplet digital (dd) PCR to measure postpartum cell-free circulating *INS* DNA levels in women following a GDM pregnancy. For this study, we used plasma obtained 12 weeks following an index GDM pregnancy, and women were followed for up to 10 years for the development of T2DM.

## 2. Methods

### 2.1. Participant Recruitment and Sample Collection

The Mercy Health Human Research Ethics Committee approved the study, and written informed consent was obtained from all participants. The women were participants in the GDM follow-up program, and details have been published previously [18–20]. Twelve-week postnatal blood samples were collected in EDTA tubes, centrifuged at 1,000 g for 10 min; plasma was supplemented with 0.1 mmol/l phenylmethylsulfonyl fluoride protease inhibitor (USB, Cleveland, OH) and immediately stored at -80°C until assayed as detailed below.

### 2.2. Measurement of Cell-Free Circulating *INS* DNA

DNA was isolated, processed, and analyzed following the method described by Fisher et al. [12]. Briefly, DNA was extracted from 50 *μ*l of plasma (frozen unthawed aliquots) of the participants using the QIAamp DNA blood mini kit (QIAGEN) with 10 *μ*g poly-A as a carrier. Of the extracted DNA, 20 *μ*l (250-350 pg/*μ*l) was then bisulfite converted using the EZ DNA Methylation-Lightning kit (Zymo Research), which converts >99.5% of nonmethylated C residues to U, while protecting >99.5% of methylated cytosines. Droplets were generated using the automated droplet generator, and bisulfite-converted DNA was analyzed by droplet digital PCR (ddPCR, Bio-Rad) using a dual fluorescent probe-based multiplex assay. The selected probe [12] could distinguish DNA that is differentially methylated at bp -69 of the human insulin gene. Samples were analyzed using a QX200 Droplet Reader and the QuantaSoft Analysis Pro Software (Bio-Rad), to determine the concentration (copies/*μ*l) of unmethylated and methylated *INS* DNA. Plasmids for insulin cfDNA (unmethylated, methylated, or combinations of these) were used on each assay plate as positive controls. Inter- and intra-assay CVs were less than 5%. The total cell-free insulin DNA (copies/*μ*l) represents the sum of the copies of unmethylated as well as methylated insulin DNA from each sample.

### 2.3. Statistical Analysis

Statistical analyses were performed using the SPSS software (v22), and significance was assigned when *P* values were less than 0.05. Characteristics of the NGT women and women with T2DM were compared using the Mann-Whitney *U* test. For the cell-free *INS* DNA data, data were log transformed and compared using an unpaired Student *t*-test. Spearman's correlation was used to assess the relationship between maternal BMI and age and cell-free *INS* DNA.

## 3. Results

**Figure 1:**
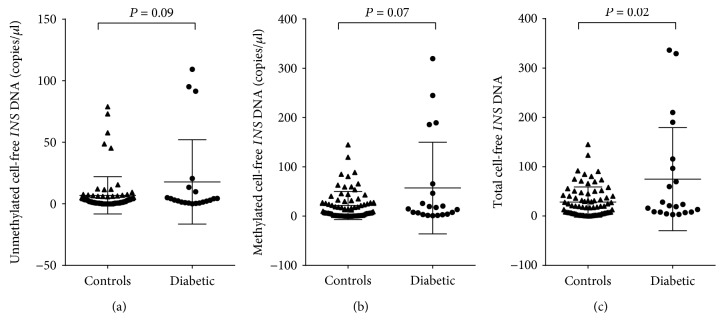
Postpartum circulating cell-free *INS* DNA levels in women with a previous GDM pregnancy. Droplet digital PCR was used to measure levels of cell-free circulating methylated (a) and unmethylated (b) *INS* DNA in plasma from 97 women, 12 weeks following an index GDM pregnancy. After 10 years, 76 women remained NGT (controls; NGT) while 21 women developed T2DM (T2DM). (c) Cell-free total *INS* DNA levels represent the sum of the unmethylated and methylated *INS* DNA levels. Data are presented as mean ± sd.

Samples from a total of 97 women were used for this study, and the clinical details are as described in Table 1. The median follow-up period was 8.7 years, and during this time, 76 participants remained NGT while 21 developed T2DM. The median time to the development of T2DM was 5.8 years. Cell-free circulating unmethylated and methylated *INS* DNA levels were not different between NGT women and women who developed T2DM (Figures 1(a) and 1(b)). On the other hand, compared with NGT women, total cell-free *INS* DNA levels were significantly higher in women who developed T2DM (Figure 1(c)). There was no significant relationship between maternal age or maternal BMI and cell-free circulating unmethylated and methylated *INS* DNA or total cell-free *INS* DNA levels.

## 4. Discussion

Elevations of cell-free circulating unmethylated *INS* DNA correlate with dying *β*-cells in both mice and humans [7, 9, 11, 13–15]. In this study, we used ddPCR to determine absolute copy numbers of cell-free circulating *INS* DNA levels in plasma obtained 3 months postpartum from women with an index GDM pregnancy. In this cohort of women, we measured circulating unmethylated and methylated *INS* cfDNA between women with a history of GDM who developed T2DM and those who remained NGT during the 10-year follow-up period. While cell-free unmethylated and methylated *INS* DNA were higher in women who progressed to T2DM, they did not reach statistical significance (*P* = 0.11 and *P* = 0.07, respectively). Total cell-free *INS* DNA levels, however, which represent the sum of unmethylated *INS* DNA and methylated *INS* DNA, were significantly higher in women who progressed to T2DM compared to those that remained NGT.

We have previously demonstrated that postpartum plasma C-peptide is positively associated with the development of T2DM in women with previous GDM [19]. C-peptide is a small peptide that is cleaved from proinsulin in the synthesis of active insulin and reflects both physiologic insulin secretion and insulin released from damaged or dead *β*-cells. Increase in unmethylated *INS* cfDNA is thought to arise mainly from pancreatic *β*-cells, and it is thought that this DNA is released into circulation when islet *β*-cells are destroyed during disease progression [8]. In our study, however, we did not see any difference in cell-free unmethylated *INS* DNA in T2DM progress versus T2DM nonprogress suggesting no *β*-cell death. There are a few possible reasons for this discrepancy. Firstly, the -69 site was chosen for these analyses as this site is preferentially unmethylated in islet *β*-cells [8, 9] and is very stable to changes in metabolic stress such that it does not undergo methylation in T2DM [21]. Analyzing multiple unique sites, such as by using sequencing technologies, may possibly improve the assay specificity but is sample and cost prohibitive. Secondly, we analyzed samples only at one time point (~3 months postpartum) which may miss events that occur over time. Indeed, when compared to NGT women, women with a previous GDM pregnancy have evidence of reduced *β*-cell function five years postpartum [17].

In contrast to unmethylated *INS* DNA, methylated *INS* DNA can arise from any nonislet *β*-cell tissue [8, 9]. In our study, postpartum methylated *INS* cfDNA increased in peripheral circulation of women who subsequently developed T2DM. The reason for this increase is not known. Interestingly, however, in an experimental animal model of type 1 diabetes, the levels of methylated *INS* DNA in *β*-cells increased as a direct result of proinflammatory stressors [10]. It is unclear as to what may be the source of methylated insulin cfDNA. Fisher et al. [12] indicate that the most likely and convincing source of methylated cfDNA in their cohort of individuals with or at risk of type 1 diabetes is from autoreactive T-cells. Although autoimmunity is not involved in GDM, several studies [22] and a meta-analysis [23] point to an increase in proinflammatory cytokines in GDM vs. non-GDM pregnant mothers. Even though the exact source of methylated cfDNA remains unknown, the increase in total insulin cfDNA, found in our study, may reflect this increased proinflammatory cytokine profile and/or other systemic effects of hyperglycemia during pregnancy.

This is the first report analyzing postpartum insulin cfDNA in a longitudinal study cohort of women with GDM (*n* = 97). In an earlier cross-sectional study, 22 women with GDM were compared with 14 non-GDM pregnant women with an aim to assess the death of pancreatic *β*-cell (increased unmethylated insulin cfDNA) in the women with GDM [24]. These investigators did not observe any increased islet *β*-cell death in mothers with GDM compared to those without GDM. Our measurements were postpartum and with an aim to understand if increased cellular death in the immediate postpartum period was an indicator of future diabetes. We did not observe any increase in unmethylated insulin cfDNA copies in women who progressed to T2DM during the study. This is in line with the gestational islet *β*-cell death measurements made by Kenna et al. [24], suggesting that there is no islet *β*-cell death during pregnancy or immediately postpartum but that the overall effect of GDM-related stressors affects nonislet tissues that possibly induce a series of inflammatory events resulting in reduced *β*-cell function and progression to T2DM later in life.

It is interesting to note that it appears from the data that there are two subgroups in the group of women who develop T2DM: those that have increased levels of cell-free *INS* DNA and those that do not. To our knowledge, there is nothing different about the early postpartum characteristics of the women with the high cell-free *INS* DNA levels. Further, the women who remained NGT were on average younger and less obese than those who developed T2DM. There was, however, no significant relationship between maternal age or BMI and cell-free circulating unmethylated and methylated *INS* DNA or total cell-free *INS* DNA levels.

A study strength is that the increased cellular death assessed in terms of circulating cfDNA is associated with the development of T2DM during the next 2-9 years. Further studies involving longitudinal analyses of circulating cfDNA from mothers with GDM are merited. The limitations of the study have been discussed previously [18–20] and include a moderately small sample size, lack of normal pregnant women as controls, and limited information on how many of the women in each group had additional pregnancies and what proportion of these were affected by GDM. Further insufficient clinical information on the participants meant we were unable to make adjustments for ethnicity or family history of diabetes. Notably, there is a different risk for post-GDM development of T2DM for women of different ethnicities. Therefore, future studies must be performed using a larger and well-characterised cohort of patients in order to assess if cell-free circulating *INS* DNA can be used to predict the development of T2DM in women with a previous GDM pregnancy. These studies should also be designed to include normoglycemic pregnant women who did and did not develop T2DM postpartum. These studies will also help to inform the values required for inclusion for long-term follow-up in terms of risk stratification.

In conclusion, in this cohort of women with previous index GDM pregnancy, postpartum levels of total cell-free circulating *INS* DNA are significantly higher in women who go on to develop T2DM. In order to determine the predictive value of cell-free *INS* DNA for the development of T2DM, a larger study population is required.

## Figures and Tables

**Table 1:** Characteristics of the women used in this study.

Characteristics	T2DM	NGT	*P* value^1^
	(median and IQR)	(median and IQR)	
	(*n* = 21)	(*n* = 76)	
OGTT during pregnancy			
Fasting (mmol/l)	5.3 (4.7-5.7)	4.7 (4.4-5.3)	**0.008**
1 hour (mmol/l)	10.2 (9.7-11.4)	9.7 (8.6-11.0)	0.140
2 hour (mmol/l)	9.1 (8.1-10.8)	8.6 (8.1-9.3)	0.302
Postnatal; time of sample collection			
Age (years)	35.1 (31.6-37.7)	31.8 (29.2-34.89)	**0.007**
BMI (kg/m^2^)	29.1 (23.4-34.7)	24.7 (22.6-28.1)	**0.024**
Fasting OGTT (mmol/l)	5.0 (4.8-5.3)	4.6 (4.4-4.9)	**0.001**
1 hour OGTT (mmol/l)	7.2 (6.3-8.6)	6.2 (5.3-7.6)	**0.030**
2 hour OGTT (mmol/l)	5.3 (4.8-6.2)	5.2 (4.4-6.0)	0.313
Cholesterol	6.0 (5.0-7.1)	5.2 (4.6-6.0)	**0.011**
Triglycerides	1.1 (0.8-1.5)	0.9 (0.6-1.2)	**0.043**

NGT: normal glucose tolerant; OGTT: oral glucose tolerance test; IQR: interquartile range. ^1^*P* values are computed using the Mann-Whitney test.

## Data Availability

The authors confirm that the data supporting the findings of this study are available within the article.

## Additional Points

*Highlights*. Women with gestational diabetes have a sevenfold higher risk of future type 2 diabetes (T2DM). The present study demonstrates for the first time that postpartum circulating cell-free insulin DNA levels are significantly higher in women with gestational diabetes and could be used to stratify those at risk of progressing to T2DM in later life.

## References

[1] Chen L., Magliano D. J., Zimmet P. Z. (2012). The worldwide epidemiology of type 2 diabetes mellitus—present and future perspectives. *Nature Reviews Endocrinology*.

[2] Shaw J. E., Sicree R. A., Zimmet P. Z. (2010). Global estimates of the prevalence of diabetes for 2010 and 2030. *Diabetes Research and Clinical Practice*.

[3] Lee A. J., Hiscock R. J., Wein P., Walker S. P., Permezel M. (2007). Gestational diabetes mellitus: clinical predictors and long-term risk of developing type 2 diabetes: a retrospective cohort study using survival analysis. *Diabetes Care*.

[4] Ratner R. E., Christophi C. A., Metzger B. E. (2008). Prevention of diabetes in women with a history of gestational diabetes: effects of metformin and lifestyle interventions. *The Journal of Clinical Endocrinology & Metabolism*.

[5] Schwarzenbach H., Hoon D. S. B., Pantel K. (2011). Cell-free nucleic acids as biomarkers in cancer patients. *Nature Reviews Cancer*.

[6] Antonatos D., Patsilinakos S., Spanodimos S., Korkonikitas P., Tsigas D. (2006). Cell-free DNA levels as a prognostic marker in acute myocardial infarction. *Annals of the New York Academy of Sciences*.

[7] Zhang K., Lin G., Han Y., Xie J., Li J. (2017). Circulating unmethylated insulin DNA as a potential non-invasive biomarker of beta cell death in type 1 diabetes: a review and future prospect. *Clinical Epigenetics*.

[8] Kuroda A., Rauch T. A., Todorov I. (2009). Insulin gene expression is regulated by DNA methylation. *PLoS One*.

[9] Husseiny M. I., Kaye A., Zebadua E., Kandeel F., Ferreri K. (2014). Tissue-specific methylation of human insulin gene and PCR assay for monitoring beta cell death. *PLoS One*.

[10] Rui J., Deng S., Lebastchi J., Clark P. L., Usmani-Brown S., Herold K. C. (2016). Methylation of insulin DNA in response to proinflammatory cytokines during the progression of autoimmune diabetes in NOD mice. *Diabetologia*.

[11] Lebastchi J., Deng S., Lebastchi A. H. (2013). Immune therapy and *β*-cell death in type 1 diabetes. *Diabetes*.

[12] Fisher M. M., Watkins R. A., Blum J. (2015). Elevations in circulating methylated and unmethylated preproinsulin DNA in new-onset type 1 diabetes. *Diabetes*.

[13] Herold K. C., Usmani-Brown S., Ghazi T. (2015). *β* cell death and dysfunction during type 1 diabetes development in at-risk individuals. *The Journal of Clinical Investigation*.

[14] Husseiny M. I., Kuroda A., Kaye A. N., Nair I., Kandeel F., Ferreri K. (2012). Development of a quantitative methylation-specific polymerase chain reaction method for monitoring beta cell death in type 1 diabetes. *PLoS One*.

[15] Fisher M. M., Perez Chumbiauca C. N., Mather K. J., Mirmira R. G., Tersey S. A. (2013). Detection of islet *β*-cell death in vivo by multiplex PCR analysis of differentially methylated DNA. *Endocrinology*.

[16] Gala-Lopez B. L., Neiman D., Kin T. (2018). Beta cell death by cell-free DNA and outcome after clinical islet transplantation. *Transplantation*.

[17] Lekva T., Bollerslev J., Godang K. (2015). *β*-cell dysfunction in women with previous gestational diabetes is associated with visceral adipose tissue distribution. *European Journal of Endocrinology*.

[18] Lappas M., Jinks D., Shub A., Willcox J. C., Georgiou H. M., Permezel M. (2016). Postpartum IGF-I and IGFBP-2 levels are prospectively associated with the development of type 2 diabetes in women with previous gestational diabetes mellitus. *Diabetes & Metabolism*.

[19] Lappas M., Jinks D., Ugoni A., Louizos C. C. J., Permezel M., Georgiou H. M. (2015). Post-partum plasma C-peptide and ghrelin concentrations are predictive of type 2 diabetes in women with previous gestational diabetes mellitus. *Journal of Diabetes*.

[20] Lappas M., Mundra P. A., Wong G. (2015). The prediction of type 2 diabetes in women with previous gestational diabetes mellitus using lipidomics. *Diabetologia*.

[21] Yang B. T., Dayeh T. A., Kirkpatrick C. L. (2011). Insulin promoter DNA methylation correlates negatively with insulin gene expression and positively with HbA_1c_ levels in human pancreatic islets. *Diabetologia*.

[22] Abell S. K., De Courten B., Boyle J. A., Teede H. J. (2015). Inflammatory and other biomarkers: role in pathophysiology and prediction of gestational diabetes mellitus. *International Journal of Molecular Sciences*.

[23] Xu J., Zhao Y. H., Chen Y. P. (2014). Maternal circulating concentrations of tumor necrosis factor-alpha, leptin, and adiponectin in gestational diabetes mellitus: a systematic review and meta-analysis. *The Scientific World Journal*.

[24] Kenna L. A., Olsen J. A., Spelios M. G., Radin M. S., Akirav E. M. (2016). *β*-cell death is decreased in women with gestational diabetes mellitus. *Diabetology & Metabolic Syndrome*.

